# The Nerve Growth Factor Metabolic Pathway Dysregulation as Cause of Alzheimer’s Cholinergic Atrophy

**DOI:** 10.3390/cells11010016

**Published:** 2021-12-22

**Authors:** Sonia Do Carmo, Benjamin Kannel, A. Claudio Cuello

**Affiliations:** 1 Department of Pharmacology and Therapeutics, McGill University, Montreal, QC H3G 1Y6, Canada; sonia.docarmo@mcgill.ca; 2 Department of Neurology and Neurosurgery, McGill University, Montreal, QC H3A 2B4, Canada; benjamin.kannel@mail.mcgill.ca; 3 Department of Anatomy and Cell Biology, McGill University, Montreal, QC H3A 0C7, Canada; 4 Department of Pharmacology, Oxford University, Oxford OX1 3QT, UK

**Keywords:** nerve growth factor, NGF metabolic cascade, cholinergic system, Alzheimer’s disease, Down syndrome, basal forebrain cholinergic nuclei, trophic support

## Abstract

The cause of the loss of basal forebrain cholinergic neurons (BFCNs) and their terminal synapses in the cerebral cortex and hippocampus in Alzheimer’s disease (AD) has provoked a decades-long controversy. The cholinergic phenotype of this neuronal system, involved in numerous cognitive mechanisms, is tightly dependent on the target-derived nerve growth factor (NGF). Consequently, the loss of BFCNs cholinergic phenotype in AD was initially suspected to be due to an NGF trophic failure. However, in AD there is a normal NGF synthesis and abundance of the NGF precursor (proNGF), therefore the NGF trophic failure hypothesis for the atrophy of BCNs was abandoned. In this review, we discuss the history of NGF-dependency of BFCNs and the atrophy of these neurons in Alzheimer’s disease (AD). Further to it, we propose that trophic factor failure explains the BFCNs atrophy in AD. We discuss evidence of the occurrence of a brain NGF metabolic pathway, the dysregulation of which, in AD explains the severe deficiency of NGF trophic support for the maintenance of BFCNs cholinergic phenotype. Finally, we revise recent evidence that the NGF metabolic dysregulation in AD pathology starts at preclinical stages. We also propose that the alteration of NGF metabolism-related markers in body fluids might assist in the AD preclinical diagnosis.

## 1. Introduction

This review deals with some general historical aspects regarding the fascinating discovery of nerve growth factor (NGF). It tells how this trophic factor was first found to be significant for the survival of basal forebrain cholinergic neurons (BFCNs) during developmental stages. It also relates how NGF was later established as responsible for the maintenance of their neuronal phenotype in the adult and fully differentiated central nervous system (CNS).

For decades, it was assumed that mature NGF (mNGF) was generated intracellularly from its precursor molecule (proNGF) and released in an activity-dependent manner as the mature form. This assumption stemmed from studies performed in vitro and applying ELISA methods unable to distinguish mNGF from proNGF. However, investigations made ex vivo with cerebral cortex slices stimulated with carbachol, glutamate, or potassium chloride revealed only the activity-dependent release of proNGF (not mNGF) along with the release of a cluster of zymogens, convertases, and endogenous regulators composing a metabolic cascade responsible for the extracellular maturation and degradation of NGF. This metabolic pathway, discovered by us and referred to as the NGF metabolic cascade, is described in detail in this review along with its marked dysregulation in the Alzheimer’s disease (AD) pathology.

We discuss here how this metabolic dysregulation results in reduced mNGF generation and its exacerbated degradation provoking a trophic void for the maintenance of highly NGF-dependent BFCNs. We also discuss the evidence of an early, preclinical, NGF metabolic dysregulation in the continuum of the AD pathology in sporadic cases as well as in Down syndrome (DS) with preclinical AD pathology.

Finally, this review discusses the possible causative effect of CNS amyloid burden and inflammation for this AD-pathology-related NGF metabolic dysregulation. It also points to the potential value of studying levels of members of this metabolic cascade in body fluids (cerebrospinal fluid (CSF) and plasma) to assist in the early detection of preclinical AD pathology.

## 2. NGF as the First Nervous System Trophic Factor

For decades, embryologists wondered if the growth of limbs was controlled by the nervous system or the reverse. Viktor Hamburger’s finding that the extirpation of the wing bud in chick embryos prevented the development of the CNS led him to advance the idea that “The wing stimulates the central nervous system by way of a reflex arc and that only its sensory fields are the acting parts. They send nervous excitations centripetally to the spinal ganglia” [1]. This paper and methodology profoundly inspired a young Rita Levi-Montalcini to further investigate the developmental relationship of peripheral tissue and the nervous system. Investigations were initiated with outstanding mentor Giuseppe Levi and were carried out clandestinely when the “racial laws” were dictated in Italy during World War 2. These studies provoked a publication (that went rather unnoticed during those war years) that contradicted Hamburger’s view [2]. However, this and subsequent post-war papers of Levi-Montalcini posed the possibility that peripheral tissues modulated nervous system development. This provoked an invitation from Viktor Hamburger to spend a year in St Louis to resolve the dispute, a collaboration that was extended and led to the discovery of NGF. In 1948, while investigating the impact of a variety of tumors on the developing nervous system, Elmer Bueker, Hamburger’s former graduate student, found that a mesenchymal sarcoma stimulated neural growth and concluded that “sarcoma 180, because of intrinsic physicochemical properties and mechanics of growth, selectively causes the enlargement of spinal ganglia” [3]. Inspired by this finding, Levi-Montalcini and Hamburger then provided indisputable evidence that peripheral sarcoma 180 provoked neuronal growth via a diffusible factor [4,5,6]. The biochemical characterization of this factor was propelled by the participation of biochemist Stanley Cohen by demonstrating the proteinaceous nature of “nerve growth stimulating factor” [7,8], and by discovering that snake venom and rodent submaxillary gland had high contents of “nerve growth factors” inducing growth of primary sensory neurons [9]. That chain of discoveries opened the field of growth factors and permitted Angeletti and Bradshaw to decipher the amino-acid sequence of NGF [10], and later to solve its crystal structure revealing its dimeric organization consisting of three antiparallel pairs of beta-strands [11].

The purification of NGF in large amounts then allowed for the derivation of antibodies against NGF. With these tools in hand, Levi-Montalcini demonstrated that NGF is necessary for the development, growth, and maintenance of the sympathetic and sensory systems, thus establishing NGF as the first neurotrophic factor to be identified [9,12,13], a discovery further substantiated by Levi-Montalcini’s lab and others [14,15,16,17]. These discoveries facilitated the finding of a new family of NGF-like trophic factors acting in the nervous system and referred to as “neurotrophins”, as well as the identification of defined receptors responding to these ligands, as reviewed in [18,19,20,21,22].

In the 1980s, it was demonstrated that NGF also acts on the development of cholinergic neurons of the rat CNS where it increases the activity of choline acetyltransferase (ChAT), the enzyme responsible for the generation of acetylcholine, both in vitro and in vivo [23,24,25,26,27,28]. Further, intraventricular administration of NGF increased ChAT activity in the developing basal forebrain of neonatal rats [24,28,29]. NGF also increased ChAT activity and promoted survival and fiber growth in neuronal cultures prepared from fetal rat septum [23,24,27,30,31]. It was also shown that NGF protein and transcript levels paralleled the growth of cholinergic neurons projections in the developing rat brain [32,33,34,35,36].

Importantly, the application of NGF and of anti-NGF antibodies in vitro indicated that the embryonic survival and development of BFCNs are highly dependent on the expression of both NGF and its receptors tropomyosin receptor kinase A (TrkA) and p75NTR [37,38], further cementing the importance of NGF as a trophic factor in the survival and development of BFCNs in the developing mammalian CNS.

## 3. Role of NGF in the Maintenance of the Cholinergic Phenotype of Basal Forebrain Neurons and Synapses in the Adult CNS

That NGF is critical for the survival and maintenance of the phenotype of specific subsets of peripheral neurons and basal forebrain cholinergic nuclei during development and maturation is quite clear. The idea that NGF is essential for the survival of BFCNs is largely based on the in vitro studies cited above.

There is also strong evidence showing that NGF acts as a neurotrophic factor for cholinergic neurons of the **adult** CNS. The selective uptake and retrograde transport of NGF from the dorsal hippocampus to the adult forebrain cholinergic system was first shown by Schwab and collaborators [39]. However, the specific physiological effects of this observation were unknown at that time. A few years later, it was shown that levels of NGF and its mRNA are highest in areas of the CNS which are rich in basal forebrain cholinergic projections, such as the hippocampus and cerebral cortex [40,41,42].

The concept that NGF remains as a “survival” factor in the adult brain emerged from the protective effects of exogenously administered NGF in models of axotomy of the fimbria fornix which disconnects the cholinergic neurons of the medial septum from the hippocampus, where the target-derived neurotrophin is largely produced. Such axonal transection results in the disappearance of ChAT-immunoreactive neurons of the medial septum. This neuronal loss can be prevented by the intraventricular application of exogenous mature NGF protein and was therefore interpreted as an NGF-dependent survival mechanism [43,44,45]. These investigations created much interest in the remarkable trophic effects of NGF, creating an expectation on the possible recovery of the forebrain cholinergic system in AD. They were based on the assumption that depriving the BFCNs of their NGF supply at the terminal targets induced “cell death” and that NGF had a “survival” effect, as referred to in the above pioneering publications. However, Sofroniew and collaborators demonstrated that extensive stroke-like lesions of the cerebral cortex involving the synaptic terminals of axonal projections originating from cholinergic neurons of the nucleus basalis did not induce cellular death but rather neuronal atrophy [46]. The further demonstration that the medial septum cholinergic neurons survived after the excitotoxic elimination of hippocampal target neurons, reinforced the notion that, in the septal-hippocampal lesion model, the cause of cholinergic neuronal death is the actual fimbria-fornix lesion [47].

The above sequence of investigations strongly supports the concept that diminished NGF trophic support does not provoke cholinergic neuronal cell death but rather progressive retrograde atrophy of these neurons [46,48,49]. The same concept can be extended to the human species. In the human brain, the neurons of the cholinergic nucleus basalis of Meynert (nbM) are “magnocellular” (i.e., notably larger neurons). The Science communication of Whitehouse and collaborators [50] reporting nbM neuronal loss, in AD, was based on observations applying Nissl staining techniques and, therefore, the “disappearance” of large neurons in this nucleus was interpreted as cell death [50,51]. The use of immunohistochemical techniques, however, showed that, in AD, the number of ChAT-immunoreactive cells in the nucleus basalis remains stable but that neurons undergo neuronal atrophy rather than cell death [46,48,49,52].

As discussed above, NGF supply appears to have a definitive effect on the survival of BFCNs in in vitro conditions and during development. A strong argument for the critical role of NGF in the developmental survival of these neurons has been provided by Chen and collaborators [53] as the loss of one NGF allele in mice led to a decreased number of cholinergic neurons in the medial septum. However, the position of our lab is that, in the adult and fully differentiated nervous system, NGF plays an important role in the phenotypic maintenance of the neuronal and synaptic cholinergic phenotype of BFCNs rather than in their survival [54,55,56]. Importantly, in the adult CNS, NGF exerts a positive regulation of its own high affinity receptor [57,58,59,60,61,62]. There is also well-established evidence that NGF controls the expression of housekeeping proteins, transcription factors, ribosomal proteins as well as cytoskeletal proteins responsible for anterograde and retrograde axonal transport [63,64,65,66,67,68]; therefore, such losses in BFCNs can simply be attributed to a deficiency in NGF trophic support, as discussed in the subsequent section.

The efficacy of exogenous NGF in recovering lesion-induced atrophy of BFCNs has been strongly demonstrated by many laboratories, as reviewed in [69,70,71,72,73]. That evidence has provoked the therapeutic possibilities of the clinical application of *exogenous* NGF in AD, a subject of continued interest and discussed in authoritative reviews [74,75,76]. On the other hand, we pose the possibility that improving the stable and constant brain level of *endogenous* NGF should be a more promising avenue. The existence of a CNS metabolic pathway regulating the available mature NGF, as discussed below, might offer hitherto unknown therapeutic possibilities. This objective is also substantiated by the evidence that the trophic support offered by minute amounts of endogenous mature NGF is sufficient to regulate the day-to-day number of NGF-sensitive cholinergic synapses in the cerebral cortex [57]. A finding which is in line with the classical Hebbian hypothesis of activity-dependent synaptic growth and enhanced CNS neuronal assemblies [77]; fundamental ideas which led to the widely accepted concept of “synaptic plasticity”.

## 4. Does a Metabolic NGF Dysregulation Explain the Cholinergic Atrophy in the Alzheimer’s Pathology?

### 4.1. The BFCN Compromise in AD Pathology

Much of the interest in the neurobiology of trophic maintenance of BFCNs has been provoked by the well-established notion that this system plays a fundamental role in higher CNS functions and memory [78], and subsequently by the discovery of the early and significant loss of cortical cholinergic biochemical markers in the AD pathology [79,80], followed by the cholinergic hypothesis of memory loss in aging proposed by Bartus and collaborators [81]. These studies, along with the reported “loss” of cholinergic neurons of the nbM [82], accelerated clinical, preclinical, and pathological investigations on the forebrain and gave rise to the so-called “cholinergic hypothesis of Alzheimer’s disease” [83].

Altogether, the above studies led to the development and application of anticholinesterases in AD, which for decades have remained the most widely applied strategy for symptomatic treatment [84,85,86]. However, while initially effective in preserving cognition, the effects of anticholinesterase treatment are of limited duration as the maintenance of the “cholinergic tone” is dependent on the presence of the remaining telencephalic cholinergic synapses which are gradually lost with the disease progression, despite anticholinesterase treatment. The recent realization that the degeneration of BFCNs in AD predicts the atrophy of brain regions innervated by their projections, such as the entorhinal cortex and cerebral cortex [87,88,89,90], is a further invitation to considering the best strategies to protect the NGF-dependent BFCNs from their unavoidable atrophy as the AD pathology progresses.

### 4.2. The NGF Metabolic Cascade

**Figure 1:**
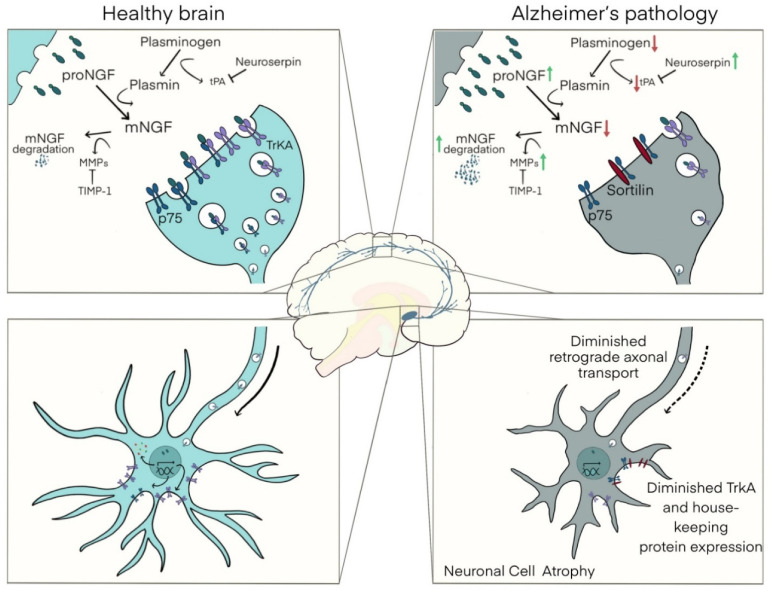
The NGF pathway and its dysregulation in Alzheimer’s and Down syndrome pathology. In the healthy brain (**left**), upon neuronal stimulation, proNGF is secreted into the synaptic cleft along with the zymogens and convertases involved in its maturation and degradation. proNGF is converted to mNGF by plasmin, itself produced from the cleavage of plasminogen by tPA, under the control of neuroserpin. mNGF then dimerizes and binds to p75/TrkA receptor complexes on presynaptic terminals of BFCNs, to be internalized and retrogradely transported to their neuronal soma in the basal forebrain to fulfill its trophic functions. These include the control of the expression of housekeeping proteins, transcription factors, ribosomal proteins, and cytoskeletal proteins responsible for axonal transport. Receptor-unbound mNGF is rapidly degraded by metalloproteinases, produced under the control of TIMP-1. In the brain of individuals with Alzheimer’s pathology (**right**), including those with Down syndrome, increased neuroserpin and decreased tPA limit the conversion of plasminogen into plasmin. As plasmin is responsible for the maturation of proNGF to mNGF, this results in a build-up of proNGF. In parallel, increased MMP-9 and MMP-3 and decreased TIMP-1, their natural inhibitor, resulted in the excessive degradation of free, receptor-unbound mNGF. This two-pronged attack on the NGF metabolic cascade, driven by AD pathology, leads to a substantial decrease in mNGF bioavailability, and thus a substantial decrease in the trophic support of BFCNs, leading to their atrophy. Arrows indicate the direction of the alterations for each important member of the NGF pathway. Red represents a reduction in the protein levels and green an elevation.

A new investigative paradigm regarding the NGF trophic support of BFCNs is presently available with the discovery of a metabolic pathway controlling the availability of mNGF as well as its extracellular degradation [91] (Figure 1). The discovery of the NGF pathway resolved the paradox that, in the brain of AD sufferers, BFCNs degenerate while the levels of NGF transcripts remain unchanged [92,93,94] and the protein levels of the NGF precursor, proNGF, are greatly elevated [94,95,96,97,98,99,100].

In brief, mNGF is produced transiently in the synaptic cleft via an NGF metabolic cascade in which proNGF, zymogens, convertases, and endogenous regulators are co-secreted from BFCN-target neurons in an activity-dependent manner [91]. Upon the extracellular release of these factors, the inactive zymogen plasminogen is cleaved into its active enzyme form plasmin by tissue plasminogen activator (tPA), in a process regulated by the tPA inhibitor, neuroserpin [91]. Plasmin is then responsible for the cleavage of the prodomain of proNGF resulting in the production of mature NGF and the N-terminus domain of proNGF, of unknown function. Degradation of the remnants of the receptor-unbound mNGF is accomplished by the metalloproteases MMP-3 and MMP-9. Their precursors, proMMP-9 and proMMP-3, are released from the BFCN target cells alongside tissue inhibitor of metalloproteinases-1 (TIMP-1) which regulates the cleavage of these precursors into their mature forms [91,101] (Figure 1).

Our lab has validated this pathway pharmacologically, demonstrating that applying neuroserpin or blocking plasmin activity compromises the conversion of proNGF to mature NGF leading (as in the AD pathology) to increased proNGF brain levels, atrophy of BFCNs, and loss of cholinergic synapses [91,102,103]. Therefore, we posit that it is the dysregulation of the NGF metabolic cascade that underlies the BFCN dysfunction in AD.

That hypothesis is supported by the finding that an NGF metabolic dysregulation is indeed present in the AD pathology even at preclinical stages as demonstrated in the analysis of post-mortem brain tissue, plasma, and CSF [96,98,100,104,105,106]. In the AD brain, NGF dysmetabolism is defined by a compromise of proNGF maturation, and a parallel excessive degradation of mNGF [106,107,108]. This compromise of the NGF metabolic cascade leads to a substantial decrease in the bioavailability of mNGF, and thus a substantial decrease in the trophic support of BFCNs, leading to neuronal cell atrophy and loss of synapses at their terminal sites.

We have shown that the postmortem frontal cortex tissue from AD patients has notably increased protein levels of neuroserpin, the endogenous tPA inhibitor, leading to decreased tPA protein levels and activity resulting in lower plasmin brain content [98,106,109]. These changes in NGF maturation explain the well-established build-up of proNGF in AD brains [94,95,96,97,98,99,100], a build-up caused by the unaltered proNGF synthesis and failure of conversion to mNGF.

The AD NGF metabolic dysregulation is a double-edged sword, as not only is NGF maturation compromised, but also the degradation of the already diminished mNGF is intensified, due to a substantial elevation of MMP-9 and MMP-3 activity [98,104,110]. Importantly, and paralleling the recent brain imaging findings of a preclinical atrophy of the basal forebrain area [87,88,89,90], we have found evidence that the NGF metabolic dysregulation starts at preclinical stages [106]. In this regard, we have recently found that individuals with no cognitive impairment (NCI) but with high brain β-amyloid (Aβ) burden (HA-NCI) demonstrate heightened levels of proNGF, plasminogen, and MMP3 and reduced levels of tPA [106]. This evolving NGF dysmetabolism correlated with cerebral Aβ and Tau deposition, cognitive performance, and loss of cholinergic synapses [106].

### 4.3. NGF Dysmetabolism in Down Syndrome

A similar imbalance of the NGF pathway has been evidenced in the brains of individuals with DS at preclinical AD stages [108,110,111,112,113,114]. Due to the triplication of the 21st chromosome, which contains the APP gene locus, DS individuals are a well-established high-risk population for AD. In fact, DS is now regarded as the most common form of genetic AD, and AD presentation in DS (DSAD) is similar to that of autosomal-dominant AD (ADAD) [115,116,117,118]. By mid-life DS sufferers inevitably develop advanced AD pathology [119,120,121], which makes DS cohorts invaluable for the investigation of AD biomarker trajectories. Confirmation of the value of this population can be seen in the initiation of various AD biomarker investigative projects such as the Alzheimer’s Biomarkers Consortium-Down Syndrome (ABC-DS, https://www.nia.nih.gov/research/abc-ds, accessed on 2 December 2021) [122] and the Down Alzheimer Barcelona Neuroimaging Initiative (DABNI, https://santpaumemoryunit.com/alzheimer-down-unit/, accessed on 2 December 2021) and in longitudinal clinical studies [123,124].

In DS brains, proNGF accumulation is accompanied by increases in neuroserpin protein levels, TIMP1 transcripts and MMP-9 activity as well as by decreases in plasminogen protein levels and in tPA transcripts [108,110,111,112,113,114]. Therefore, NGF dysmetabolism should account for the AD-like cholinergic atrophy observed in DS brains [125,126,127]. Notably, such dysregulations are already present in primary cultures from fetal DS cortex displaying high levels of amyloid peptides [108,128].

The NGF metabolic dysregulation has also been replicated in the brain of transgenic rodent models of the human AD-like amyloid pathology [98,129,130] as well as in the Ts65Dn mouse model of DS [108], a model displaying increased APP protein and transcript levels [131,132,133,134,135,136,137,138].

In consequence, we hypothesize that the NGF metabolic dysregulation in the continuum of the AD pathology, resulting in the abrogation of trophic support to the NGF-dependent BFCNs, is ultimately the cause of their well-established atrophy in this neurodegenerative condition. We further postulate that the NGF trophic failure leading to a progressive degeneration of BFCNs makes them vulnerable to tau pathology [139,140]. The same situation would apply to the loss of TrkA receptors in the BFCNs and in their terminals [141,142,143,144,145] as NGF signaling is required for the expression of its high affinity receptor, TrkA, as well as for all the NGF-dependent expression of housekeeping proteins, including those related to axonal transport, as discussed above.

The consequences of a compromised NGF metabolic pathway on BFCNs in the AD pathology are schematically represented in Figure 1.

### 4.4. A Link between Amyloid Pathology, Inflammation, NGF Dysregulation, and Basal Forebrain Cholinergic Atrophy

**Figure 2:**
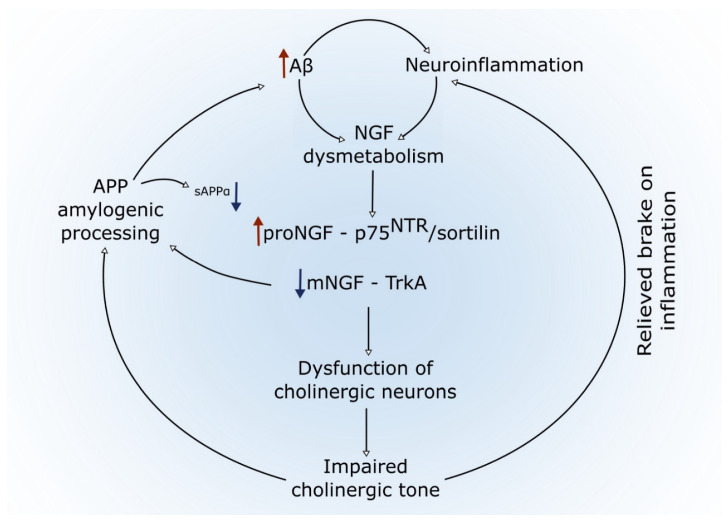
The proposed self-perpetuating cycle links amyloid pathology, inflammation, NGF dysregulation, and basal forebrain cholinergic atrophy. The accumulation of toxic Aβ peptides oligomers produced from the amyloidogenic processing of APP induces an early neuroinflammatory process as well as early NGF dysmetabolism. The latter results in an excess of proNGF and depletion of mNGF and a coincident regulation of their specific receptors. Aβ-driven overproduction of proinflammatory mediators can also itself lead to a dysregulation of the NGF pathway. The reduced bioavailability of mNGF then leads to diminished cholinergic trophic support resulting in BFCNs’ atrophy and loss of function. In turn, the reduced cholinergic tone further promotes amyloidogenic processing of APP, as well as neuroinflammation, given the suggested inflammatory suppressive effects of acetylcholine. Together, this leads to a self-perpetuating cycle promoting further dysfunction of the cholinergic system.

The NGF dysmetabolism in the AD pathology is likely to be provoked by progressive Aβ burden and other pathological factors of which CNS inflammation is an important candidate (Figure 2). The tight association between amyloid pathology, inflammation, and NGF dysmetabolism is supported by several lines of research. In favor of this concept is the finding that injection of Aβ oligomers in the hippocampus of naïve rats provoked both brain inflammation and NGF dysregulation and that this NGF dysmetabolism was rescued via the application of an anti-inflammatory compound [98]. It is also of particular interest that matrix metalloproteases, including MMP-9, which participates in the NGF metabolic pathway, have a significant role in pro-inflammatory pathways [146,147,148]. Moreover, Aβ is known to induce strong inflammatory responses in the context of progressive amyloid pathology [149,150,151]. There is also experimental evidence that the basal forebrain cholinergic system has an anti-inflammatory role in the CNS [152,153,154,155]. The association between Aβ and NGF pathway dysfunction is further strengthened by the fact that Aβ load is highly correlated with the elevation of proNGF in older DS individuals, and that changes to mNGF pathway proteins correlate within tissues to amyloid load in AD [106,112]. Such a view is further reinforced in the case of the preclinical AD stages in DS in which both biomarkers of amyloid pathology and inflammation are correlative with the degree of the NGF metabolic compromise [113]. The importance of NGF in the AD pathology is further supported by a genome-wide association study showing a strong association between a single nucleotide polymorphism (SNP) (rs9908234) located in the intron 1 of the NGF receptor gene, encoding p75NTR, and brain Aβ load as determined by positron emission tomography [156].

## 5. Will the NGF Metabolic Dysregulation Assist the Identification of Preclinical AD Stages?

The widescale acknowledgment that the neuropathological progression of AD begins decades before clinical symptom onset, and that the neurological damage present at this point is likely irreversible [157,158,159,160,161], has led to an increased interest for the identification of biomarkers capable of signaling the disease prior to when it becomes clinically apparent. A time when prevention therapy might be possible.

As high levels of proNGF and neuroserpin have been found in both CSF and plasma, reflecting the brain’s NGF metabolic dysfunction [95,96,100,105,106,110,112,162,163], it is worth considering the possibility that these NGF metabolism-based CSF and plasma biomarkers might assist in the early AD diagnosis. This possibility is supported by the fact that, as discussed above, these markers are significantly altered at AD preclinical stages both in DS [110,111,112] and in the brain of NCI individuals with AD-like amyloid burden [106]; in all cases correlative with the degree of cognitive decline. The prospect of this pathway as a source of biomarkers is furthered as a longitudinal assessment of the data set showed that an increase in proNGF levels over a 1-year interval predicted prospective cognitive deterioration over the subsequent year [111]. Additionally, other members of the NGF pathway showed an ability to differentiate DSAD from asymptomatic DS at levels similar to, or exceeding that of core AD biomarkers (tTau, pTau, ab40/42 ratio) [106]. These findings are of great significance for the potential use of NGF metabolism as a biomarker of evolving AD pathology, both in the general population and in populations at high risk of AD such as DS individuals.

## 6. Conclusions

In this review, we highlight the existence of an entire CNS metabolic pathway explaining the activity-dependent release of proNGF along with a cluster of molecules which, in a highly coordinated fashion, provoke the conversion of the NGF precursor molecule to its mature and trophic active form in the extracellular space, in proximity to their cognate receptors located in cholinergic synaptic terminals. The mNGF rapidly binds to these receptors to be internalized and retrogradely transported to the neuronal soma to fulfill its trophic functions. The function of the remnant unbound mNGF in the extracellular space is terminated by the proteolytic degradation effected by matrix metalloproteases.

The AD pathology impairs proNGF to mNGF conversion and facilitates mNGF degradation. This compromised NGF trophic support to BFCNs explains the well-known atrophy of these neurons in AD and it is already present at AD preclinical stages. An aspect in line with the remarkable advances in brain imaging revealing that the atrophy of the BFCN area starts at preclinical stages of the continuum of this neurodegenerative condition.

We also pose the possible contribution of alterations of body fluids biomarkers of the NGF metabolic dysregulation to signal preclinical stages of AD.

Finally, it is conceivable that pharmacological corrections of the NGF metabolic pathway could assist in preserving the trophic support of BFCNS in silent AD stages and thus prevent the atrophy of these neurons and losses of cortical and hippocampal cholinergic synapses, therefore improving cognitive outcomes.
